# Mining genomic regions associated with agronomic and biochemical traits in quinoa through GWAS

**DOI:** 10.1038/s41598-024-59565-8

**Published:** 2024-04-22

**Authors:** Hifzur Rahman, Prashant Vikram, Yulan Hu, Sugandha Asthana, Abhinav Tanaji, Padmaktshni Suryanarayanan, Chris Quadros, Lovely Mehta, Mohammed Shahid, Anestis Gkanogiannis, Sumitha Thushar, Salma Balazadeh, Bernd Mueller-Roeber, Luis Augusto Becerra Lopez-Lavalle, Tong Wei, Rakesh Kumar Singh

**Affiliations:** 1 International Center for Biosaline Agriculture, Dubai, UAE; 2 BGI-Research, 518083 Shenzhen, China; 3 Birla Institute of Technology and Science Pilani, Dubai Campus, Dubai, UAE; 4 Present Address: SGT University, Gurugram, Haryana India; 5 Institute of Biology Leiden, Sylvius Laboratory, Leiden University, Sylviusweg 72, 2333 BE Leiden, The Netherlands; 6 Department of Molecular Biology, University of Potsdam, Karl-Liebknecht-Straße 24-25, Haus 20, 14476 Potsdam, Germany; 7 BGI Research, 430074 Wuhan, China

**Keywords:** Quinoa, Abiotic stress, QTL, Saponin, Marginal environment, GWAS, LD, Agricultural genetics, Genetic association study, Agricultural genetics

## Abstract

Quinoa (*Chenopodium quinoa* Willd.), an Andean crop, is a facultative halophyte food crop recognized globally for its high nutritional value and plasticity to adapt to harsh conditions. We conducted a genome-wide association study on a diverse set of quinoa germplasm accessions. These accessions were evaluated for the following agronomic and biochemical traits: days to 50% flowering (DTF), plant height (PH), panicle length (PL), stem diameter (SD), seed yield (SY), grain diameter (GD), and thousand-grain weight (TGW). These accessions underwent genotyping-by-sequencing using the DNBSeq-G400R platform. Among all evaluated traits, TGW represented maximum broad-sense heritability. Our study revealed average SNP density of ≈ 3.11 SNPs/10 kb for the whole genome, with the lowest and highest on chromosomes Cq1B and Cq9A, respectively. Principal component analysis clustered the quinoa population in three main clusters, one clearly representing lowland Chilean accessions, whereas the other two groups corresponded to germplasm from the highlands of Peru and Bolivia. In our germplasm set, we estimated linkage disequilibrium decay to be ≈ 118.5 kb. Marker-trait analyses revealed major and consistent effect associations for DTF on chromosomes 3A, 4B, 5B, 6A, 7A, 7B and 8B, with phenotypic variance explained (PVE) as high as 19.15%. Nine associations across eight chromosomes were also found for saponin content with 20% PVE by qSPN5A.1. More QTLs were identified for PL and TGW on multiple chromosomal locations. We identified putative candidate genes in the genomic regions associated with DTF and saponin content. The consistent and major-effect genomic associations can be used in fast-tracking quinoa breeding for wider adaptation across marginal environments.

## Introduction

Quinoa (*Chenopodium quinoa* Willd.) is an Andean edible seed crop that can thrive well in areas affected by adverse impacts of climate change, particularly increasing soil and water salinity and water scarcity. Quinoa is a facultative halophyte and its most tolerant cultivars can produce economic yield even at a quite high concentration of salinity (~ 15 dS/m, which is about one-third of seawater salinity) at which other crops fail to grow. It has earned special attention worldwide because of its exceptional nutritional quality and health benefits and its ability to adapt to marginal environments, especially nutrient-poor saline soils and drought-stressed marginal agroecosystems^1–3^. In many target regions, economically viable cultivation of quinoa requires an improvement of diverse traits. Examples are a shortening of the growth period, a decrease in bitter metabolites (saponins) in grains (to improve taste), and further optimization of water use efficiency. Current varieties in the public domain often do not have the desired traits, thus discouraging farmers from growing quinoa. Furthermore, the extra costs incurred in grain processing and the lack of facilities limit the interest of farmers in cultivating quinoa. Based on these assumptions, we aim to breed desired varieties that represent high-yielding improved sweet germplasm with early maturity. This will allow poor farmers to obtain “free-to-use” sweet quinoa varieties because existing sweet quinoa varieties come from the private sector and are mostly poorly adapted to Middle East and North Africa (MENA), sub-Saharan Africa (SSA), and Central Asia and the Caucasus (CAC) regions.

Large-scale breeding activities are a challenge in quinoa because of its very-small-size florets and the presence of both hermaphrodite and female flowers (outcrossing 0.5–17%) on the same panicle^4^. Most past quinoa breeding activities had been reported from Bolivia and Peru^5,6^; however, some recent breeding studies have reported the development of high-yielding varieties adapted to temperate regions and high latitudes of Europe, North America, and China^4,7^. Conventional breeding is difficult as it takes a long time to come up with a desired variety; hence, robust molecular markers are needed to accelerate the breeding process. Molecular markers have been used in quinoa (but on a limited scale) to ascertain true crosses only^8,9^.

Recently, the first quinoa reference genome, using the coastal Chilean accession PI 614886 with a total genome size of ~ 1.5 Gb, was reported by Jarvis et al.^10^. Based on the available knowledge on genetic resources, our aim is to accelerate the breeding process of next-generation varieties. A genome-wide association study (GWAS) in quinoa was attempted using a genotyping-by-sequencing (GBS) approach^11^, and also through whole-genome sequencing, which resulted in 2.9 million markers with significant marker-trait associations identified for several agronomic traits^12^. Since our goal is to develop high-yielding, early-maturing varieties with a low saponin content in the grains, we aim at mining large-effect QTLs for the target traits and validating them, and speeding up the breeding process using marker-assisted selection (MAS). In view of this, we have chosen to develop an association mapping population to assess genetic diversity and perform a GWAS for agronomically important traits in quinoa. The mining of reliable molecular markers can solve the limitations of early selection for traits appearing at later growth stages, thus enabling fast and efficient breeding of tailored quinoa varieties with desired traits.

## Results

### Phenotypic evaluation

**Figure 1:**
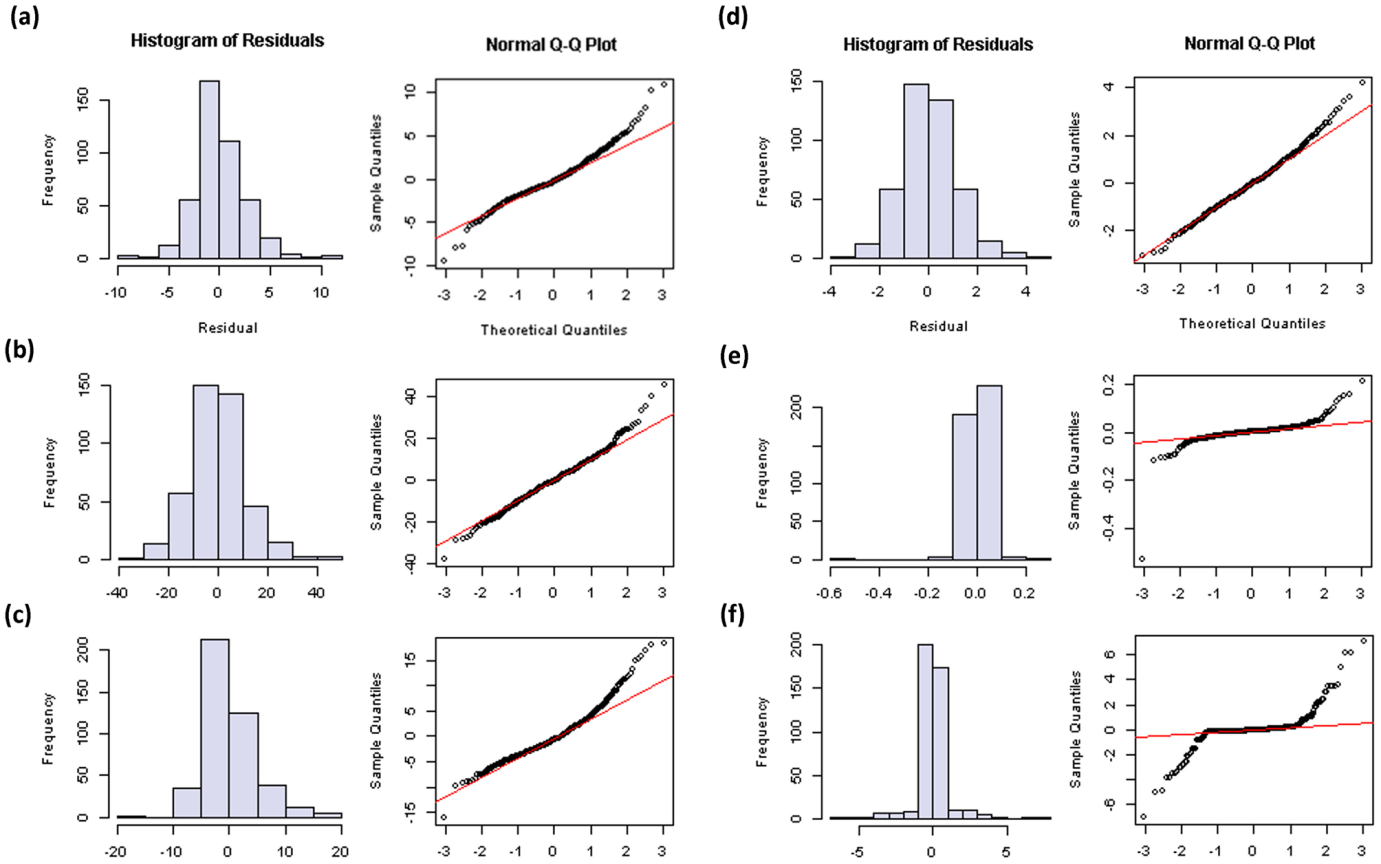
Frequency distribution, histogram of residuals, and normal Q–Q plot for various phenotypic traits: (**a**) panicle length, (**b**) plant height, (**c**) seed yield, (**d**) stem diameter, (**e**) thousand-grain weight, and (**f**) days to flowering.

The association mapping panel, mostly comprising genotypes from natural populations (Supplementary Table S1), displayed significant phenotypic variation for the traits under investigation. Table 1 presents the range, mean, standard deviation, and heritability values for different traits across two seasons. Days to flowering (DTF), plant height (PH), stem diameter (SD), panicle length (PL), seed yield/plant (SY), grain diameter (GD), and thousand-grain weight (TGW) ranged from 36 to 76 days, 24–184 cm, 2.6–15.6 mm, 7.8–51.6 cm, 1.5–73.5 g, 1.3–2.4 mm, and 0.8–4.6 g, respectively. The greatest variation was observed for plant height and seed yield, whereas grain diameter, thousand-grain weight, and days to flowering showed the lowest variation. The broad-sense heritability for all traits ranged from 46 to 99%. The heritability of SD was found to be the minimum, whereas the heritability of TGW was up to 99%. All traits showed normal or near-normal distribution among the population lines as revealed by histogram of residuals, and normal Q-Q plot (Fig. 1).

**Table 1:** Descriptive statistics, heritability, and analysis of variance of phenotypic traits for association mapping panel.

Trait	Season	Range	Mean ± SD	Heritability	F-value
DTF (days)	2019–2020	36–76	45.1 ± 6.4	0.90	12.16***
	2020–2021	37–63	44.4 ± 3.6	0.81	8.8953***
	Combined	36.3–75.1	44.9 ± 6.4	0.99	38.3727***
PH (cm)	2019–2020	24–184	86.4 ± 22.8	0.53	2.7788
	2020–2021	29.3–180.2	88.5 ± 21.5	0.65	3.698***
	Combined	50.5–154.4	87.4 ± 22.15	0.84	4.4819***
SD (mm)	2019–2020	2.6–15.6	8.4 ± 1.95	0.46	2.1121*
	2020–2021	5.0–15.2	8.9 ± 1.9	0.58	2.6897**
	Combined	6.2–11.3	8.7 ± 1.96	0.73	2.6665***
PL (cm)	2019–2020	14.0–51.6	26.2 ± 5.7	0.68	3.3665***
	2020–2021	7.8–50.6	27.8 ± 5.9	0.68	3.3724***
	Combined	20.7–37.6	26.9 ± 5.9	0.72	2.7739***
SY (g)	2019–2020	2.9–58.7	17.3 ± 9.4	0.69	3.3793***
	2020–2021	1.5–73.5	25.1 ± 11.7	0.68	3.1635***
	Combined	13.5–37.7	21.2 ± 11.3	0.52	1.8589***
GD (mm)	2019–2020	1.3–2.2	1.9 ± 0.16	0.98	52.1631***
	2020–2021	1.4–2.4	2.01 ± 0.15	0.98	60.8131***
	Combined	1.6–2.1	1.97 ± 0.25	0.43	2.9037***
TGW (g)	2019–2020	0.8–4.3	3.3 ± 0.6	0.99	106.3832***
	2020–2021	1.2–4.6	3.25 ± 0.5	0.94	22.6099***
	Combined	1.6–4.0	3.25 ± 0.68	0.69	4.9114***

DTF, days to flowering; PH, plant height; SD, stem diameter; PL, panicle length; SY, seed yield/plant; GD, grain diameter; TGW, thousand-grain weight.

*, **, *** denotes the significance at p value 0.05, 0.01 and 0.001 respectively.

### Sequencing and SNP analysis

**Figure 2:**
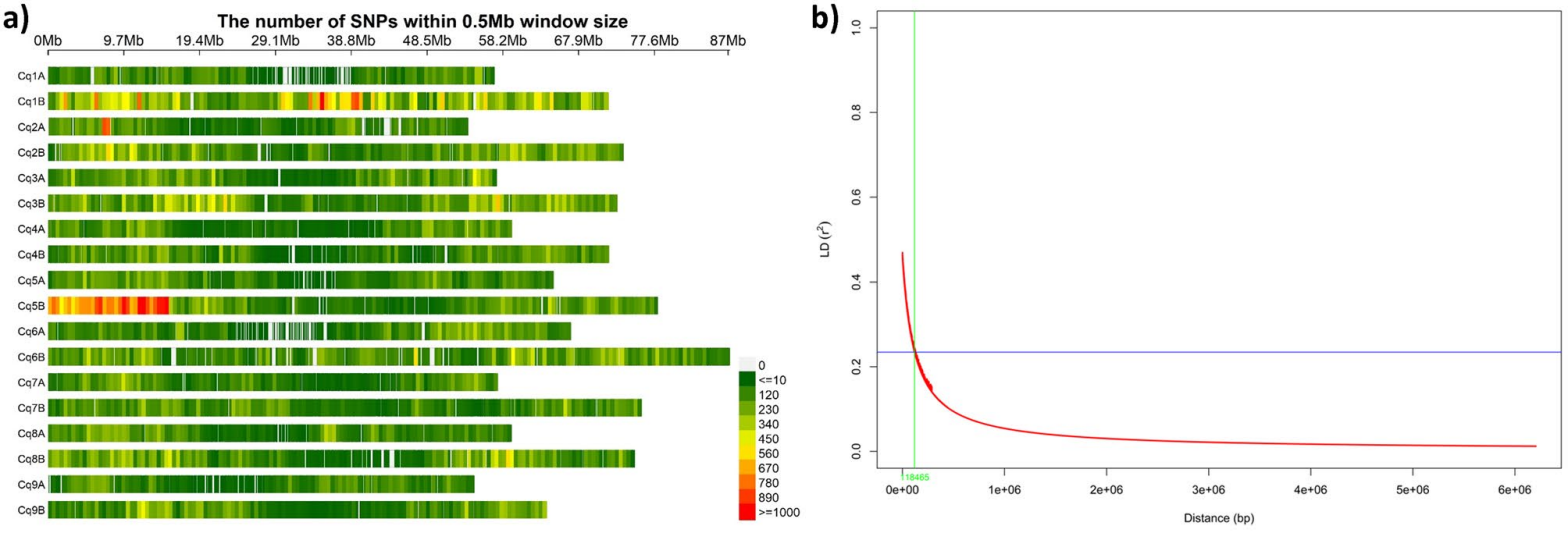
(**a**) SNP density across 18 chromosomes of quinoa representing the number of SNPs within a 0.5-Mbp window size; (**b**) genome-wide linkage disequilibrium decay of r^2^ values against physical distance (bp); the LD decay has been considered as the distance at which r^2^ dropped to half of its maximum value (0.4691).

Sequencing of 201 accessions revealed a mapping depth in the range of 6.44–15.06 X, with an average of 10.95 X, whereas the genome coverage ranged from 55.42 to 98.40%, with an average coverage of 97.11%. MAF-filtered SNPs were further filtered manually considering heterozygous loci as missing, which resulted in 383,251 high-quality SNPs. Assuming a total length of all 18 chromosomes of 1.2 Gb as reported by Jarvis et al.^10^, the average SNP density was ≈ 3.11 SNPs/10 kb, for which the highest and lowest marker densities were observed on chromosomes Cq1B (6.43 SNPs/10 kb) and Cq9A (2.10 SNPs/10 kb), respectively (Fig. 2a; Supplementary Table S2). We observed a high SNP density on the B genome (≈ 3.78 SNPs/10 kb) vis-à-vis the A genome (≈ 2.44 SNPs/10 kb). The chromosome-wise distribution of all SNPs is given in Supplementary Table S2.

### Genetic diversity analysis

**Figure 3:**
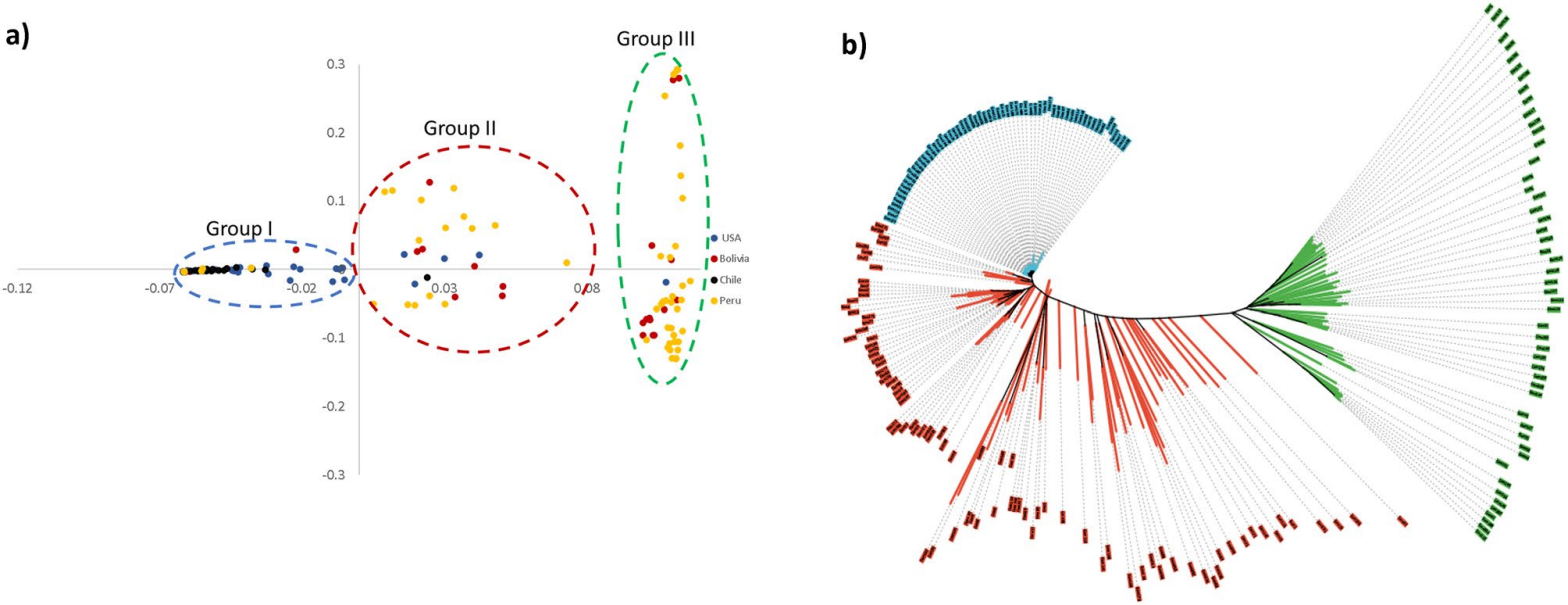
(**a**) PCA analysis; (**b**) neighbor joining tree of association mapping panel. Group I contains the genotypes from lowlands majorly, whereas group II and III were dominated by accessions that originated in highlands.

Principal component analysis clustered the quinoa population into three main clusters (Fig. 3a). The first and second principal components (PC1 and PC2) explained 37.65% and 9.42% of the total variation, respectively. Group I mostly consisted of accessions from lowlands mainly belonging to Chile, whereas group II and group III consisted of accessions from highlands belonging to Peru and Bolivia. Quinoa accessions collected by Washington State University (WSU), U.S., were found across all three groups as presented in Fig. 3a by “x”. The neighbor-joining (NJ) tree also divided the population into three clusters. One of the three clusters comprised a few accessions from Chile along with some from the U.S., while the other two clusters consisted of lines mainly from highland accessions, with a few from lowlands (Fig. 3b).

The average value of r^2^ as a function of the inter-marker distance was used for linkage disequilibrium (LD) decay calculation. LD decay in the population was determined as ≈ 118.5 kb, where the r^2^ value drops to below half of its maximum (Fig. 2b).

### Genome-wide association analyses of agronomic traits

Genomic associations were declared significant following FDR ≤ 0.1 and − Log10P ≥ 4 as a threshold with more than two SNP associations in a genomic region equivalent to the LD decay (118.5 kb). Marker-trait associations were identified for three out of seven agronomic traits studied and one biochemical trait (saponin content). Significant associations were found for DTF, PL, TGW, and saponin content, but not for PH, SD, and GD. We identified a total of 19 genomic regions associated with four traits: DTF, PL, TGW, and saponin content (Table 2, Supplementary Table S3). Genomic associations for DTF exhibited a significant effect across the two seasons as well as in the combined analysis, whereas other agronomic traits showed significant associations in either of the season and in the combined analysis also.

**Table 2:** Marker trait associations for various agronomic and biochemical traits in quinoa.

Trait	MTA name	Chr	Span (Mb)	Interval (kb)	No. of SNPs	Phenotypic variance R^2^ (%)		
						Trial 1	Trial 2	Combined
DTF	qDTF3A.1	Cq3A	17.32–17.49	196.88	4	11.9–15.4	9.47–10.6	10.39–13.91
DTF	qDTF4B.1	Cq4B.1	6.81–6.84	27.64	4	11.19	11.84	10.93
DTF	qDTF5B.1	Cq5B	9.73–9.88	152.64	25	9.00–12.67	13.85–17.01	10.59–14.26
DTF	qDTF6A.1	Cq6A	9.11–9.14	26.95	4	12.49–13.65	15.98–19.15	13.28–16.26
DTF	qDTF7A.1	Cq7A	1.93–2.07	139.79	6	12.40–13.09	13.31–14.01	12.11–12.75
DTF	qDTF7B.1	Cq7B	59.54–59.66	121.70	26	17.65–18.48	16.71–17.59	15.95–16.87
DTF	qDTF8B.1	Cq8B	23.35–23.43	77.01	4	9.91–11.15	11.55–12.67	12.07–13.26
TGW	qTGW1B.1	Cq1B	48.87–49.00	130.79	12	15.33–16.52	NS	11.21–17.79
PL	qPL3A.1	Cq3A	10.79–10.84	49.22	5	NS	16.9–22.18	10.90–21.42
PL	qPL7B.1	Cq7B	19.98–20.16	180.35	5	NS	9.70–11.5	12.65–13.97
SPN	qSPN1B.1	Cq1B	68.97–69.04	81.24	4	12.6–13.1		
SPN	qSPN3B.1	Cq3B	65.33–65.43	102.75	5	14.4		
SPN	qSPN3B.2	Cq3B	69.18–69.33	156.36	6	14.2–18.3		
SPN	qSPN4A.1	Cq4A	51.50–51.59	89.40	7	12.7–16.1		
SPN	qSPN4B.1	Cq4B	55.48–55.52	43.60	7	14.6–16.3		
SPN	qSPN5A.1	Cq5A	11.50–11.74	237.32	4	12.9–20.3		
SPN	qSPN5B.1	Cq5B	8.89–9.12	225.90	13	11.4–14.8		
SPN	qSPN8B.1	Cq8B	19.60–19.80	196.60	5	12.2–15.6		
SPN	qSPN9A.1	Cq9A	7.67–7.68	12.36	3	13.8–14.5		

Where DTF = days to flowering; TGW = thousand-grain weight; PL = panicle length; SPN represents saponin content in quinoa seeds.

Genome wide association analysis for days to flowering resulted in at total of 117 marker trait association of which 74 SNPs has shown consistent associations with either both or one of the seasons as well as in the combined analysis (Table 2, Supplementary Table S3). Theses SNPs were located across 7 genomic regions on chromosome 3A, 4B, 5B, 6A, 7A, 7B, and 8B. The genomic region qDTF3A.1 and qDTF4B.1 comprising 5 and 4 SNPs with a phenotypic variance of upto11.8 and 15.4% respectively. The DTF QTL qDTF5B.1 comprised 25 SNPs in a 152.64-kb region explaining phenotypic variance of up to 17.01%. A total of four SNPs were found associated with DTF on chromosome 6A in a 27-kb genomic region and they explained phenotypic variance of up to 19.2%. Similarly, genomic regions on chromosomes 7A (qDTF7A.1) and 7B (qDTF7B.1) revealed association with DTF and explained phenotypic variance of up to 14% and 18.5%, respectively. The numbers of SNPs in qDTF7A.1 and qDTF7B.1 regions were 6 and 26, respectively.

A major-effect QTL was also identified for TGW on chromosome 1B (qTGW1B.1) during trial 1 (2019–2020) and combined analysis using BLUP values from trial 1 and trial 2. The genomic region qTGW1B.1 spans 130.79 kb harboring 12 SNPs explaining phenotypic variance of up to 16.5% and 17.8% in trial 1 and combined analysis respectively. Furthermore, significant genomic associations were observed for PL on chromosomes Cq3A, and Cq7B, but in only trial 2 and combined analysis explaining a phenotypic variance of up to 22.18%, as presented in Table 2.

We identified nine associations across eight chromosomes (Cq1B, Cq3B, Cq4A, Cq4B, Cq5A, Cq5B, Cq8B, and Cq9A) for total saponin content (Table 2). Five large-effect associations were observed for saponin content on chromosomes 3B (qSPN3B.2), 4A (qSPN4A.1), 4B (qSPN4B.1), 5A (qSPN5A.1), and 8B (qSPN8B.1), each with a phenotypic variance of more than 15%. qSPN5A.1 explained maximum phenotypic variance (> 20%) among the five, followed by qSPN3B.2, qSPN4B.1, qSPN4A.1, and qSPN8B.1. Genomic associations were also found on chromosomes 1B, 3B, 5B, and 9A, explaining phenotypic variance in the range of 12–15% (Supplementary Table S3).

**Figure 4:**
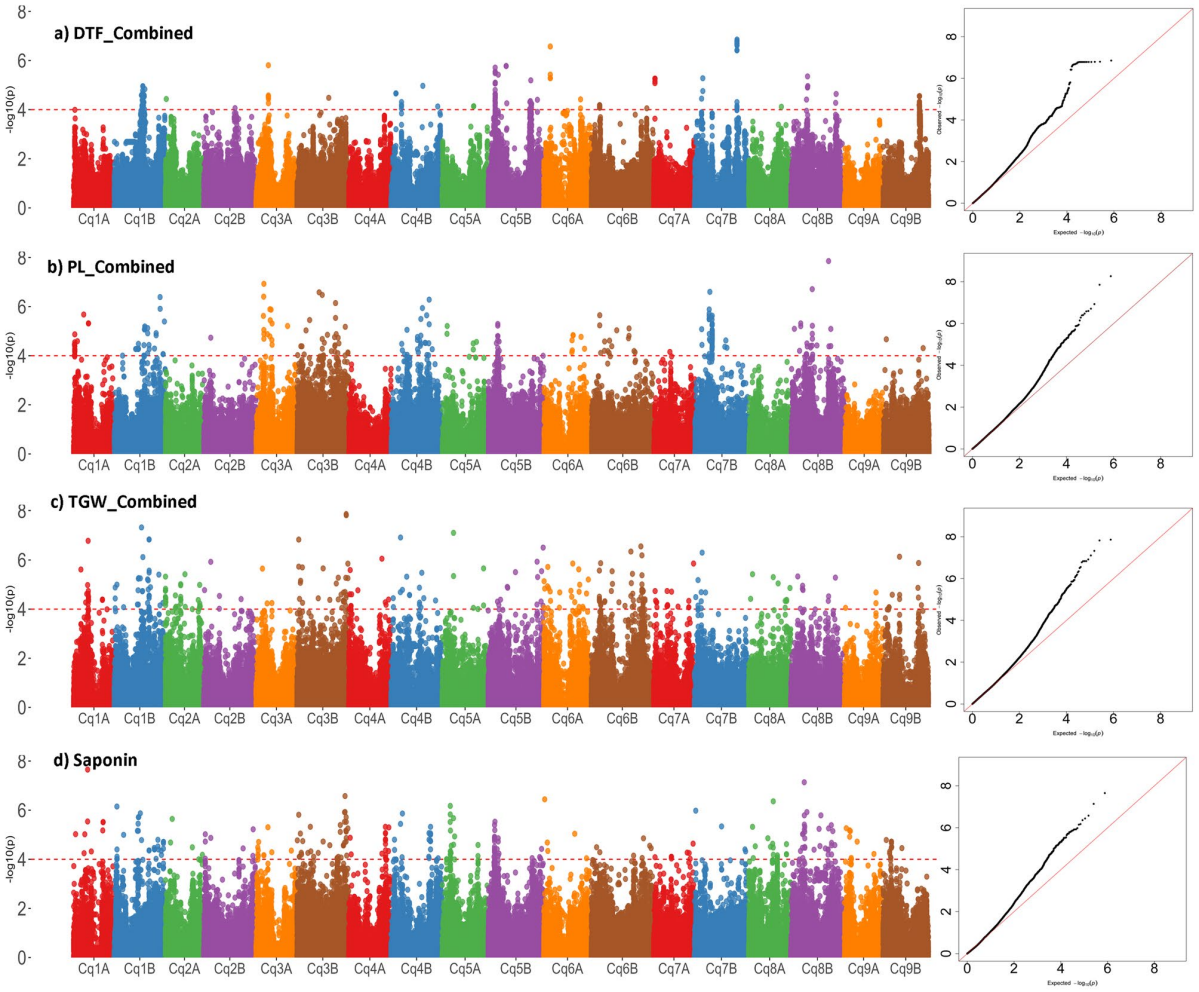
Manhattan and Q–Q plots of genome-wide association mapping of measured traits. The y-axis in each graph represents − log10P for the p-value of the MTAs, while chromosome numbers are indicated on the x-axis. The red dashed line presents the threshold − log10P value (= 4) which was used for declaring the significant associations along with another criterion i.e. number of SNPs within LD decay region.

**Figure 5:**
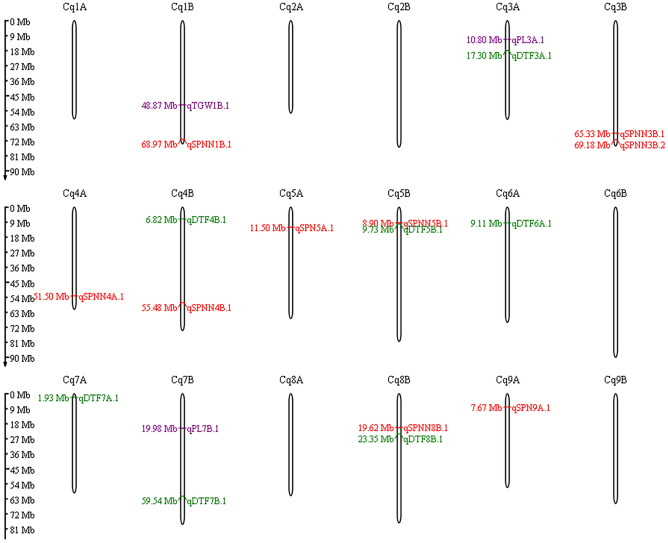
Various genomic regions identified through GWAS associated with various traits of interest. Green; purple and red text of the genomic regions indicates the genomic region identified in both individual as well as combined analysis; in either of the individual season and combined analysis and through metabolomic data respectively.

Manhattan plots depicting -log (p-values) and Q–Q plots (quantile quantile) showing the expected vs. observed p-values for the SNP-based progeny-trait of interest associations are presented in Fig. 4. Furthermore, the genomic regions on chromosomes associated with various traits are pictorially depicted in Fig. 5. Some of the chromosomes harbor more than one putative QTL for the same or different traits such as Cq3A, Cq1B, Cq3B, and Cq5B.

### Candidate genes for traits

To identify candidate genes underlying traits, we searched 118.5 kb upstream and downstream of the genomic regions associated with the traits assuming an LD decay of ~ 118.5 kb in our germplasm panel. Through GWAS analysis, we identified seven genomic regions associated with DTF. The genomic area qDTF4B.1 included 29 genes, including genes for Zinc-finger homeodomain protein 4 and PHD finger protein. The genomic region qDTF5B.1 (9.73–9.88 Mb) consisted of 39 genes, including, among others, *MYB3R5*, *FUT1*, *UGE1*, *HSFB2B*, and *CYTOCHROME P450 87A3*. Downstream of qDTF5B.1, the transcriptional regulator gene *SLK2* was located within the LD region.

The genomic region qDTF6A.1 contains a total of 21 genes (Supplementary Table S4); however, a gene encoding L10-interacting MYB domain-containing protein (*LIMYB*) within the identified genomic region was of importance for flowering. The genomic region on chromosome 7A (1.92–2.07 Mb) included *WUSCHEL-RELATED HOMEOBOX 3* (*WOX3*) gene and a gene encoding ankyrin repeat-containing protein. Apart from these genes found within the genomic region qDTF7A.1, the region downstream of the LD decay region contains a gene encoding the protein UPSTREAM OF FLC (UFC). Downstream of the genomic region qDTF8B.1 consisted of a gene encoding protein POLLENLESS 3 along with other 14 genes.

The genomic regions associated with thousand-grain weight (TGW) viz., qTGW1B.1 included gene encoding Agamous-like MADS-box protein AGL12 which was found to be of importance for grain weight. Among the two genomic regions associated with panicle length, the genomic region qPL3A1.1 included gene encoding FRL4B: FRIGIDA-like protein 4b and FAR1-RELATED SEQUENCE 7 protein whereas qPL7B.1 consists of gene encoding F-box/FBD/LRR-repeat protein.

Among nine genomic regions associated with saponin content, the genomic region qSPN5B.1 on chromosome Cq5B (8.90–9.54Mb) contained two copies of *bHLH25* and several copies of *UDP-GLYCOSYLTRANSFERASE* (*UGT*) genes. Other genomic regions associated with saponin content in quinoa seeds primarily contained genes involved in fatty acid and carbohydrate metabolism (Supplementary Table S4d).

Genes identified within the LD region of the associated genomic regions are given in Supplementary Table S4 and the major genes involved in controlling traits of interest appear in Table 3.

**Table 3:** Major candidate genes located in the identified genomic regions associated with traits of interest.

Trait	Genomic region	CoGe gene ID	Gene description	Position*	
				Start	End
DTF	qDTF4B.1	CQ020387	ZHD4: Zinc-finger homeodomain protein 4	6853293	6854009
		CQ020390	PHD finger protein	6883224	6889069
	qDTF8B.1	CQ011344	POLLENLESS 3	23517087	23520496
	qDTF5B.1	CQ004418	Transcription factor MYB3R-5	9746801	9751670
		CQ004419	FUT1-galactoside 2-alpha-l-fucosyltransferase	9753258	9755613
		CQ004421	UGE1: bifunctional UDP-glucose 4-epimerase and UDP-xylose 4-epimerase 1	9773674	9776847
		CQ004426	HSFB2B: heat-stress transcription factor B-2b	9798901	9801385
		CQ004436	CYP87A3: cytochrome P450 87A3	9911547	9914736
		CQ004442	Probable transcriptional regulator SLK2	9981368	9985459
	qDTF6A.1	CQ026113	L10-interacting MYB domain-containing protein (LIMYB)	9126463	9127853
	qDTF7A.1	CQ041079	WOX3: WUSCHEL-related homeobox 3	1935525	1938802
		CQ041113	MYB48: transcription factor MYB48	2188582	2190622
		CQ041114	UFC: Protein UPSTREAM OF FLC	2204046	2206035
TGW	qTGW1B.1	CQ023867	Agamous-like MADS-box protein AGL12	48895860	48896081
PL	qPL3A.1	CQ044917	FRL4B: FRIGIDA-like protein 4b	10820905	10825487
		CQ044935	FRS7: Protein FAR1-RELATED SEQUENCE 7	10970432	10981243
	qPL7B.1	CQ008790	F-box/FBD/LRR-repeat protein	20173766	20176099
SPN	qSPN5B.1	CQ004343	bHLH25: transcription factor bHLH25	8941949	8944828
		CQ004346	bHLH25: transcription factor bHLH25	8964690	8969517
		CQ004358	UDP-glycosyltransferase 79B30	9112292	9122129
		CQ004359	UDP-glycosyltransferase 79B30	9145388	9148852
		CQ004360	UDP-glycosyltransferase 79B30	9150011	9150250
		CQ004361	UDP-glycosyltransferase 79B30	9150292	9164132
		CQ004365	UDP-glycosyltransferase 79B30	9182896	9184631

*The position of the gene IDs refers to their position on quinoa reference genome V2 (CoGe id 60716).

Where DTF = days to flowering; TGW = thousand-grain weight; PL = panicle length; SPN represents saponin content in quinoa seeds.

## Discussion

The primary goal of our study was to identify reliable marker-trait associations for key agronomic and biochemical traits and to accelerate the development of quinoa varieties tailored for cultivation in areas with diverse environments. An early identification and selection of desirable traits, including those that appear late during plant development or at maturity, are crucial for advancing the breeding cycle. Genome-wide association studies using a diverse mapping panel offer a promising approach toward this goal because they do not require the development of permanent mapping populations (biparental or other complex populations) that consume much time.

ICBA’s gene bank has one of the largest quinoa collections outside South America. A diverse collection based on phenotypic and biochemical data was shortlisted to constitute an association mapping panel for further rigorous phenotyping over the years, and for re-sequencing the genomes of interesting quinoa accessions.

### Phenotyping

The heritability estimates, range, and Q–Q plots of the phenotypic traits in our study clearly indicate the fitness of data for marker-trait analysis (Fig. 1). The economically important trait TGW revealed the highest heritability in both seasons, suggesting its suitability in breeding programs as an important selection criterion. Heritability of DTF was found to be relatively high (81–90%), while PH showed a slightly lower heritability (53–71%) (Table 1). Most such traits are robust and relatively little influenced by genotype × environment (G × E) interaction. Maldonado-Taipe et al.^13^ also reported a high heritability of DTF (82.99%) and TGW (91.06%) in a biparental F_2:3_ mapping population derived from Chilean (PI-614889) × Peruvian (CHEN-109) accessions. Another recent study also reported high heritability of DTF (91.0%) and TGW (89.7%) in a GWAS panel comprising 303 quinoa accessions^12^. However, the heritability of the PH trait reported by Maldonado-Taipe et al.^13^ and Patiranage et al.^12^ in their respective quinoa mapping populations showed a contrasting pattern. Heritability of PH in a biparental mapping population was 38.4%^13^, whereas in a GWAS panel it was 85.0%^12^. Al-Naggar et al.^14^ also reported 60.7% heritability for PH in their studies. This moderate to high heritability of PH indicates its relatively low influence by the environment, although a more robust study involving multi-year and multi-location experiments could provide more robust information. Followed by TGW, GD also showed very high heritability, whereas the other three traits (SD, PL, and SY) revealed moderate to high heritability in both growing seasons. SD and PL also showed high correlation with PH over the seasons (70–89%), signifying the importance of all three traits for quinoa improvement programs through both direct and indirect selection (Supplementary Table S5). Nevertheless, none of the associations revealed confounding effects of PH on SD/PL or vice versa, which was surprising, suggesting that more detailed investigation is required to better understand these traits. Based on previous reports and our study, DTF and TGW are highly robust and reliable for marker-trait analysis in quinoa, followed by PH and PL. Interestingly, in our genomic association study, consistent and robust genomic regions observed over the seasons were evident only for DTF among all agronomic traits investigated.

### SNP distribution across the quinoa genome

The overall average SNP density in our study was found to be 3.11 SNPs/10 kb and the B genome had a higher SNP density than the A genome. Patiranage et al.^12^ reported an average SNP density of 2.39 SNPs/kb while performing whole-genome sequencing of 312 accessions on the Illumina platform, ranging from 0 to 122 SNPs/kb, and no significant difference was observed in SNP densities of the A and B genomes. The size of the B genome has been reported to be larger than that of the A genome in quinoa^10^. A previous report^12^ and our study clearly indicate that the genome sizes and SNP densities are not correlated. Furthermore, both studies supported the view of possible recombination and chromosomal rearrangements between the A and B sub-genomes^10^.

### Genetic diversity

In order to understand the genetic diversity of the germplasm used for our study, principal component analysis was conducted that broadly divided the whole population into three diverse groups: groups I, II, and III (Fig. 3a). Group I comprised mainly accessions from lowlands, whereas groups II and III had accessions from highlands. The majority of the lowland-specific group I accessions came from coastal areas of Chile, where temperature, light, and humidity conditions are significantly different from those of the Peruvian/Bolivian highlands. Genotypes categorized in groups II and III were mainly from the highlands of Peru and Bolivia. Recent reports with germplasm panels also divided the populations into those from highlands and lowlands, like our finding^11,12^. The first and second principal components in our study explained 37.65% and 9.42% of the total variation, respectively. In previous PCA studies^11,12^, PC1 and PC2 explained 23.35/31.6% and 9.45/6.80% variation, respectively, thereby showing a similar trend as our report. Also, similar to the analyses performed by Mizuno et al.^11^ and Patiranage^12^, accessions categorized as “collected from USA” were spread in all three sub-groups in the PC analysis in our study (Fig. 3a). This raises considerable concern about the reliability of passport data obtained from the WSU repository about the correct origin of the quinoa accessions, as addressed before^11,12^. Unlike Mizuno et al.^11^, we could not differentiate the two highland groups, even though they showed a clear distinction in Fig. 3a. This might be due to either the higher movement of germplasm materials between southern and northern highland areas or inappropriate collection site information. Similar patterns and problems were reported in a recent study on deciphering the diversity of Iranian wheat landraces^15^. A well-organized systematic germplasm collection in centers of diversity established using advanced GIS tools should help in overcoming such problems. In the case of quinoa, this is very important as researchers have defined five distinct ecotypes of *Chenopodium quinoa* based on origin^16^. However, even using high-density marker platforms, we could not explain the genetic basis of those five groups. This information is important and should be investigated in detail in the future as it could be quite useful for the improvement of quinoa germplasm and its agricultural deployment.

A mean genome-wise LD in the GWAS panel was observed at 118.5 kb estimated as decayed half to its maximum, which is one of the most commonly used thresholds to define unlinked loci^17^. Interestingly, we observed an inverse relationship between population size and LD decay in our study, which corroborates results from previous reports^11,12^. The numbers of lines in the populations of Mizuno et al.^11^, Patiranage et al.^12^, and our study were 136, 312, and 201, respectively. LD decay was found to be minimal in the population with 312 lines^12^, intermediate with 201 lines (our study), and maximal with 136 lines^11^. This clearly underpins the importance of larger population size to minimize LD decay.

### Marker-trait associations

Marker-trait associations (MTAs) largely depend on the extent of LD decay in self-pollinated crop species. There are two contrasting reports, one suggesting the non-suitability of GWAS in quinoa for determining genomic associations, while another one strongly favors it^12^. Mizuno et al.^11^ performed their study with a set of 136 genotypes phenotyped for six traits using a GBS-based assay with 5753 SNPs, whereas Patiranage et al.^12^ conducted an analysis involving 312 germplasm accessions phenotyped for 17 traits and GWAS was done with 2.9 million SNP markers identifying 1480 significant MTAs. Our MTA study was conducted on a germplasm set of 187 lines phenotyped for seven traits using 383,251 SNP markers. We successfully identified a total of 387 genomic associations with four out of seven traits investigated (Supplementary Table S3). These three reports (including our current study) highlight the importance of marker density, population size, and phenotypic as well as existing genotypic variability of the target traits.

It was interesting to note that, out of 17 traits subjected to analysis by Patinarage et al.^12^, only DTF and TGW showed a consistent effect across two seasons. Our analysis also showed the same pattern only for DTF with consistency over years. In our study, TGW also revealed moderate to high correlation with GD (Supplementary Table S5), although no significant marker-trait associations were found for GD, possibly because of the degree of precision for trait phenotyping. Phenotypic variability of GD may largely depend on the type of scale and precision used for the measurements. The higher the precision, the greater the chance for identifying QTLs. We recommend higher precision for genome-wide association studies, especially for traits with smaller dimensions such as grain diameter.

Defining MTAs in GWAS is relatively challenging as compared to biparental mapping analysis because of threshold values. Following a highly stringent and conservative criterion, Bonferroni correction, one loses the majority of the MTAs^18^, which otherwise might be declared significant considering the FDR method^19^. A large number of studies are available in which investigators define a threshold of 0.0001 (or less) to declare significant MTAs^15^. Here, we followed a slightly modified approach in which we considered FDR ≤ 0.1 and − Log_10_P ≥ 4 as a threshold only when more than one SNP was found associated in a genomic region of 118.5 kb (as per LD decay results). Interestingly, qDTF5B.1 and qDTF7B.1 revealed 19 and 33 SNPs, respectively, associated significantly with DTF within the region (Table 2; Supplementary Table S3). This method can help greatly in developing diagnostic markers and deploying GWAS results in quinoa germplasm improvement processes.

Genomic associations for saponin content were identified on chromosomes 1B, 4A, 5A, and 5B (Table 2) based on previously reported saponin content and genotypic data for our GWAS population^20^. Jarvis et al.^10^ and Patinarage et al.^12^ reported a consistent and major-effect genomic association on chromosome 5B that corroborates our findings. Patinarage et al.^12^ also reported inconsistent QTLs on chromosomes 1B, 4A, and 5A, as in our study. This suggests that these chromosomes might also harbor minor QTLs underpinning the trait. A more detailed analysis involving contrasting large biparental mapping populations would be beneficial for detecting minor QTLs with a higher confidence on these chromosomes for understanding the genetic basis of saponin content in quinoa seeds.

### Candidate genes

Putative candidate genes were searched within LD region of associated genomic regions and homologs reported for controlling flowering time or floral development within the distinct genomic regions that were identified in our study. The genomic region qDTF4B.1 consisted of genes encoding Zinc-finger homeodomain protein 4 (ZHD4) and PHD finger protein. The ZHD4 also called as *FLORAL TRANSITION AT THE MERISTEM2 (FTM2)* has been reported playing an important role in regulating floral induction in plants^21^. The PHD finger protein plays a key function in the chromatin-mediated repression of flowering, ensuring the precise control of flowering time^22,23^. It has been reported to be involved in controlling both vernalization and photoperiod pathways in Arabidopsis^24^. Another important gene near qDTF8B.1 was Protein POLLENLESS 3 which is essentials for male fertility, especially for microspore and pollen grain production in *Arabidopsis*^25^. Among the genes located in the genomic region qDTF5B.1, the *FUT1* gene encoding galactoside 2-alpha-L-fucosyltransferase regulates flower development in tobacco (*Nicotiana tabacum*)^26^ while HEAT SHOCK TRANSCRIPTION FACTOR B2b (HsfB2b) acts as a transcriptional repressor of *VIN3*, a gene induced by long-term cold for flowering in *Arabidopsis thaliana*^27^. Further downstream of qDTF5B.1, a gene encoding transcriptional regulator SLK2 was located within the LD region. SLK2 functions together with co-regulator SEUSS in LEUNIG-regulated processes during flower development, thereby controlling flowering time^28^. The genomic region qDTF7A (1.92–2.07 Mb) includes *WUSCHEL-RELATED HOMEOBOX 3* (*WOX3*) and a gene encoding an ankyrin repeat-containing protein. *WOX3* genes are involved in the formation of lateral sepals and sepal margins in flowers^29^. Although ankyrin repeat-containing protein in quinoa has, to our knowledge, not been reported to be involved in flowering control, *Arabidopsis* plants with a mutated ankyrin repeat-containing protein showed delayed flowering^30^. The genomic region downstream of qDTF7A contained a gene encoding the protein UPSTREAM OF FLC (UFC). The *UFC* gene is adjacent to *FLOWERING LOCUS C* (*FLC*), which responds to vernalization and acts as a floral repressor in many temperate species^31,32^.

The genomic region qTGW1B.1 mapped for thousand-grain weight consists of 14 genes in the LD region. However, one gene encoding Agamous-like MADS-box protein AGL12 was of importance as it has been reported to be involved in numerous developmental process including seed and embryo development^33^. The genomic regions associated with panicle length consists of genes encoding FRL4B: FRIGIDA-like protein 4b and FAR1-RELATED SEQUENCE (FRS) 7 protein in genomic region qPL3A.1 and F-box/FBD/LRR-repeat protein in qPL7B.1. The FRIGIDA-like protein and FAR1 proteins has been involved in controlling flowering time, leading to late flowering phenotype in plants and inducing the development of enlarged panicles^34,35^. Further, FRS members has been reported as the candidate genes for *Panicle and Spikelet Degeneration (PSD)* gene in rice^36^ and has been mapped in qPL5 of rice^37^. F-box protein encoding genes has been reported to be involved in floral transition as well as panicle and seed development^38^.

The genomic region qSPN5B.1 on chromosome Cq5B (8.90–9.54 Mb) associated with saponin content contains two copies of *bHLH25* and several *UGT* (*UDP-GLYCOSYLTRANSFERASE*) genes. The bHLH25 transcription factor has been identified in several studies and reported as TRITERPENE SAPONIN BIOSYNTHESIS ACTIVATING REGULATOR 2 (*TSAR2*) controlling the production of saponin in quinoa seeds^10,12,13^. Apart from *bHLH25*, the genomic region qSPN5B.1 also contains several *UDP-GLYCOSYLTRANSFERASE* genes involved in the glycosylation of triterpenoid saponins^39–42^. The other genomic regions associated with saponin content in quinoa seeds primarily contain genes involved in fatty acid and carbohydrate metabolism.

The SNP markers and candidate genes identified in this study can be efficiently used to develop the molecular markers-based robust and reliable selection system that can further help to fasten the pace of conventional breeding efforts. This is crucial for the traits which appear only during late growth stages (flowering duration and plant height) or only after harvesting (saponin content). Early identification of desired segregants through molecular markers will allow us to keep only positive plants without maintaining full population that include unwanted genotypes and save the limited resources. Similarly crossing of the desired plants would be possible without waiting for the trait to appear during late growth stage. The developed improved quinoa genotypes will be easily accepted by farmers once the major bottlenecks in quinoa genotypes (saponin content and long duration) will be overcome.

Quinoa has earned special attention worldwide due to its exceptional nutritional quality and health benefits and its ability to adapt to marginal environments especially saline soils and drought stressed marginal agroecosystems. Most of the target regions need improvement of traits like shortening of growth period, sweet grain types, reduced water requirement and non-lodging type sturdy stem with medium plant height. Current varieties in the public domain do not have these desired traits hence farmers are discouraged from cropping quinoa either due to non-availability of preferred varieties or extra cost incurred on post-harvest processing. Based on these assumptions, we targeted to breed the desired varieties for the region which are high yielding, sweet genotype with early maturity. This will allow poor farmers to have access ‘free to use’ sweet quinoa varieties as all existing sweet quinoa varieties are from private sector and mostly poorly adapted to relatively hot regions.

## Methods

### Plant material and growth conditions

The gene bank of the International Center for Biosaline Agriculture (ICBA) (https://www.biosaline.org/about-icba/facilities/genebank) contains more than 1000 *Chenopodium* spp. accessions. We previously evaluated a random set of 500 quinoa accessions for phenology at the ICBA farm during 2016–2017 and 470 out of 500 were analyzed for their triterpenoid saponin content using LC-MS^20^. To ensure a representative sample for our study, we selected 201 genotypes from the initial set of 500. We selected them based on inclusion of the full diversity spectrum available for all traits and diverse origins in a way that the panel represented all collection sites. Supplementary Table S1 provides information about the collection site of each accession in our study.

Of the 201 genotypes, 187 that had germinated and given consistent phenotypic data across trials were used for association analysis, whereas all 201 accessions were used for diversity studies and estimating LD decay (Supplementary Table S1).

### Field experiments

Field experiments were conducted at the research farm facilities of the ICBA (latitude 25.0949; longitude 55.3899), located in Dubai, United Arab Emirates. The GWAS panel was phenotyped for various morphological traits across two seasons, 2019–2020 and 2020–2021. Because of the large number of accessions, we used an augmented randomized complete block design with five blocks, involving 190 test entries and six checks to compensate for the block effect. Each accession was assigned a plot size of 1.5 × 2.5 m^2^. Weather data were collected by a meteorological station during both growing seasons and are summarized in Supplementary Table S6.

Prior to sowing, we added and mixed poultry manure (Al Yahar organic manure, UAE) at an amount of 30 t/ha. Drip irrigation was employed maintaining a plant-to-plant spacing of 25 × 25 cm. For sowing, we used the dibbling method with a depth of 1–2 cm, with three to five seeds sown next to each dripper. Three weeks after sowing, the plants were thinned to maintain a single plant per dripper. Regular hand weeding was carried out to remove weeds and off-type plants.

### Phenotypic and biochemical evaluation

Seven morphological traits—days to flowering (DTF), plant height (PH), main panicle length (PL), stem diameter (SD), seed yield per plant (SY), grain diameter (GD), and thousand-grain weight (TGW) were recorded to assess the variation among the quinoa genotypes; some of the data were previously reported^20^. DTF was recorded when approximately 50% of the plants had flowered. PH, PL, and SD were measured at the time of harvesting. SY was recorded after threshing the harvested plants and GD was determined using ten seeds, with their length averaged by dividing the total length by 10. TGW was determined by counting and weighing 1000 seeds using a seed counter. Basic statistical parameters and analysis of variance (ANOVA) were used with PBTools v1.4 (http://bbi.irri.org/products). The following linear model was used to determine the observed response of the i-th treatment in the j-th block:

$${\text{Y}}_{{{\text{ijt}}}} = \, \mu \, + {\text{ T}}_{{\text{i}}} + \, \beta_{{\text{j}}} + \, \left( {{\text{block}}:{\text{trial}}} \right) \, + \, \left( {{\text{genotype}}:{\text{trial}}} \right) \, + {\text{ e}}_{{{\text{ijt}}}}$$

Y_ijt_ is response variable in equation where µ denotes the overall mean, T_i_ denotes the effect of the genotypes, β_j_ denotes the effect of the jth block, block within trial, genotype within trial and e_ijt_ denotes the error variance of the ith treatment in the jth block of tth trial. Best linear unbiased predictions (BLUP) of the traits were calculated for all traits through PBTools v1.4 (http://bbi.irri.org/products) using one stage multi-environment analysis.

The data for the total seed saponin content of 176 quinoa genotypes were used from Tabatabaei et al.^20^ as the sum of intensity of 37 triterpenoid saponins that were detected through liquid chromatography–mass spectrometry (LC–MS) analysis.

### Genome sequencing

DNA was extracted from flash-frozen leaf samples using a modified CTAB method^43^. The purity and quality of the isolated DNA were verified by agarose gel electrophoresis on 0.8% agarose gels and the concentration was determined on a Qubit 4 fluorometer using Qubit Broad Range Assay Kit (Thermo Fisher Scientific Inc., Waltham, MA, USA). One microgram of genomic DNA was mechanically fragmented to an average size of 250 bp using the Covaris® M220 Focused-ultrasonicator™ (Covaris, Woburn, MA, USA) and the size selection of fragmented DNA was done using MGIEasy DNAClean beads (MGI Tech, Shenzhen, China). A single-stranded circular DNA library was prepared using MGIEasy Universal DNA Library Prep Set Ver. 1.0 following the manufacturer's standard protocol for a 250-bp insert size, followed by DNA nano ball (DNB) formation based on rolling circle amplification. The DNB was loaded into the flow cell (DNBSEQ-G400RS Sequencing Flow Cell Ver. 3.0) and cPAS-based 100-bp paired-end sequencing was performed with DNBSEQ-G400RS High-Throughput Sequencing Set Ver. 3.1 (MGI Tech).

### Read mapping and variant calling

To analyze the genomic DNA extracted from flash-frozen leaf samples, the sequence reads were mapped to the quinoa reference genome V2^44^ (CoGe id 60716) and SNP and indel calling was performed using Sentieon software (version 202010) (https://www.sentieon.com/). First, raw reads were filtered with Trimmomatic (version 0.39)^45^ with the parameters of “ILLUMINACLIP: adapter.fa:2:35:4:12:true, LEADING:3 TRAILING:3 SLIDINGWINDOW:5:15 MINLEN:50.” Next, the filtered reads were mapped to the quinoa reference genome (CoGe id 60,716, https://genomevolution.org/coge/GenomeInfo.pl?gid=60716) using BWA-MEM^46^ from the Sentieon software. The resulting BAM files were sorted and PCR duplicates were marked using Dedup, and then variants were called using Haplotyper in the GVCF mode. All GVCF files were then merged into one file and variants were called using GVCFtyper. Raw SNPs and indels were filtered using the Variant Filtration function in the Genome Analysis ToolKit (GATK, version 4.1.8.1) with the parameter “QD < 2.0; FS > 60.0; MQ < 40.0; SOR > 3.0; MQRankSum < -12.5; ReadPosRankSum < -8.0” for SNPs, and the parameter “QD < 2.0; FS > 200.0; SOR > 10.0; MQRankSum < -12.5; ReadPosRankSum < − 8.0” for indels^4,47^. For further population analysis, only biallelic SNPs with a missing rate of < 10% and a minor allele frequency (MAF) of > 0.05 were selected using VCFtools (version 0.1.13)^48^. For association analysis, the SNPs were further filtered by considering heterozygous alleles as missing and filtering the SNPs to a missing rate of < 10% to obtain 383,251 high-quality SNPs, which were used for the marker-trait association analysis.

### Population analysis

Principal component analysis was performed using the filtered SNP set with GCTA software (version 1.93.2 beta). A neighbor-joining tree was constructed on the same SNP set using PHYLIP (version 3.69)^49^ and the tree layout was generated using online tool iTOL (https://itol.embl.de).

### Linkage disequilibrium and GWAS analysis

The LD was determined by calculating the squared correlation coefficient (r^2^) for all manually filtered SNPs. The pairwise LD values were determined in TASSEL 5 (ver. 20230519) and the values were plotted against physical distance (bp) in R Studio following Remington et al.^50^. The pattern of LD decay was determined as the distance at which LD values declined to half of their maximum (0.4691) value. The SNP density plot was created using R package CM-plot (https://github.com/YinLiLin/CMplot) to look at the number of SNPs within 0.5-Mb windows in all 18 chromosomes.

Genome-wide association analysis was conducted with high-quality filtered SNPs using a mixed linear model. Marker-trait association was examined in TASSEL 5 (ver. 20230519) (https://www.maizegenetics.net/tassel). The first five principal components were incorporated as covariates in the GWAS model as a fixed effect whereas kinship among the genotypes was incorporated as a random effect to consider the population stratification and control false positives in the marker-trait analysis. Kinship (K) was determined using the Centered IBS (identity by state) method^51^. Analysis to establish marker-trait association was carried out for seven agronomic traits (from two consecutive trials) and saponin content in seeds of individual genotypes^20^ using a mixed linear model through TASSEL 5 (ver. 20230519). The combined GWAS analysis was further carried out using BLUP values calculated from the trait values collected from both the years (Supplementary table S7a). Manhattan plots and Q–Q plots of each trait were drawn using the modified R package “qqman”^52^.

We calculated false discovery rate (FDR) with the Benjamini and Hochberg method on a per-chromosome basis and selected SNPs with FDR < 0.1 as significant. Only those genomic regions in which multiple SNPs (three or more) showed associations with − log10 (p-value) > 4 and FDR value < 0.1 within the LD region for either of the traits were declared to be significantly associated. The group of significantly associated SNPs based on their p-values across trials was identified as putative genomic regions associated with traits and was considered as a QTL for that trait. Chromosomal map of identified MTAs/QTLs was performed using the web tool MG2C (http://mg2c.iask.in/mg2c_v2.1/) to represent their physical position on *C. quinoa* chromosomes^53^.

Putative candidate genes were manually searched within the LD region of each associated region on the quinoa reference genome (v2, id 60716) using JBrowse on the CoGe database (https://genomevolution.org/coge/GenomeView.pl?embed=&gid=60716). For the identified significant marker-trait associations, candidate gene prediction was performed within the LD decay region, that is, 118.5 kb upstream and 118.5 kb downstream of the SNPs with significant association signals. Consequently, we identified candidate genes that might be associated with the traits studied based on the LD decay in the region and their putative functions after a thorough review of the literature.

### Plant guideline statement

All the experiments which have been done comply with relevant institutional, national, and international guidelines and legislation. Seed materials used in this study has been obtained from the gene bank of International Center for Biosaline Agriculture which is a global public goods under ITPGR article 15 (https://www.biosaline.org/about-icba/facilities/genebank).

## Summary and conclusions

The genomic region discovered in our study provides an opportunity to develop robust molecular markers for marker-assisted selection (MAS) to facilitate the development of superior quinoa genotypes through crossing programs and it will help breeders in developing tailor-made early-maturing and high-yielding sweet quinoa genotypes. Further research has begun focusing on validating the discovered QTLs and characterizing candidate genes through biparental contrasting mapping populations to develop diagnostic markers in the next step. Overall, the findings of our study provide the groundwork for future research on the molecular mechanisms controlling several agronomic traits, including flowering, in quinoa. Furthermore, they will facilitate MAS in quinoa breeding programs. The marker-trait associations identified in our study will be helpful in allele mining in quinoa germplasm collections to find novel genetic variation.

### Supplementary Information

Supplementary Table S1.

Supplementary Table S2.

Supplementary Table S3.

Supplementary Table S4.

Supplementary Table S5.

Supplementary Table S6.

Supplementary Table S7.

## Data Availability

All re-sequencing data are submitted to SRA under project id BioProject PRJNA1028353.
