# Supplementary material for: Mining genomic regions associated with agronomic and biochemical traits in quinoa through GWAS
Source: Sci Rep. 2024 Apr 22;14:9205. doi: 10.1038/s41598-024-59565-8 (PMC11035704; doi:10.1038/s41598-024-59565-8)
Supplement: Supplementary file 6 — Supplementary Table S6. [file 41598_2024_59565_MOESM6_ESM.docx]

**Supplementary Table S6.** Temperature, relative humidity, and rainfall at the trial site during the cropping season. All experiments were carried out in the fields of ICBA, at geographic location of 25° 05.847 N; 055° 23.464 E, Dubai.

| Year | Month | Temperature mean (℃) | Maximum temperature mean (℃) | Minimum temperature mean (℃) | Relative humidity mean (%) | Precipitation (mm) | PAR  (W/m^2^) | Wind speed (m/s) |
| --- | --- | --- | --- | --- | --- | --- | --- | --- |
| 2019 | October | 31.21 | 41.35 | 23.58 | 43.94 | 0 | 96.27 | 2.38 |
| 2019 | November | 24.19 | 34.95 | 16.11 | 51.50 | 26.37 | 77.98 | 2.13 |
| 2019 | December | 21.34 | 29.51 | 13.36 | 60.56 | 15.82 | 66.14 | 2.14 |
| 2020 | January | 18.16 | 29.96 | 9.15 | 61.56 | 105.47 | 72.66 | 2.83 |
| 2020 | February | 20.15 | 33.90 | 10.33 | 61.06 | 5.27 | 90.91 | 2.54 |
| 2020 | March | 22.68 | 33.86 | 13.40 | 55.31 | 26.37 | 106.72 | 3.02 |
| 2020 | April | 28.91 | 41.92 | 16.12 | 43.88 | 10.55 | 110.75 | 2.82 |
|  |  |  |  |  |  |  |  |  |
| 2020 | October | 29.56 | 40.90 | 19.19 | 37.94 | 0 | 101.97 | 2.25 |
| 2020 | November | 25.58 | 35.22 | 18.05 | 55.38 | 0 | 80.95 | 2.38 |
| 2020 | December | 21.14 | 32.35 | 13.79 | 56.88 | 5.27 | 72.9 | 2.23 |
| 2021 | January | 18.65 | 29.39 | 10.12 | 52.25 | 0 | 80.59 | 2.38 |
| 2021 | February | 21.82 | 32.99 | 13.07 | 57.81 | 0 | 92.45 | 2.27 |
| 2021 | March | 25.62 | 39.05 | 13.50 | 42.94 | 0 | 111.47 | 3.18 |
| 2021 | April | 29.86 | 41.18 | 17.23 | 34.00 | 1.40 | 126.64 | 2.68 |
